# Morphological Criteria for Staging Near‐Hatching Embryos of the Domesticated Mallard (*Anas platyrhynchos*) and Swan Goose (*Anser cygnoides)*

**DOI:** 10.1002/jmor.70027

**Published:** 2025-01-29

**Authors:** Bassel Arnaout, Kaylen Brzezinski, Benjamin Steventon, Daniel J. Field

**Affiliations:** ^1^ Department of Earth Sciences University of Cambridge Cambridge UK; ^2^ Department of Genetics University of Cambridge Cambridge UK; ^3^ Department of Biology Carleton University Ottawa Ontario Canada; ^4^ Museum of Zoology University of Cambridge Cambridge UK

**Keywords:** Anseriformes, duck, embryonic development, goose, staging table

## Abstract

Studying avian embryology necessitates reliable and precise staging tables—descriptions of embryonic features appearing during development that are used to approximate the extent of embryonic development from fertilisation to hatching. Staging tables for waterfowl (Anseriformes) have previously been established based on morphological features from fertilisation to approximately 10 days before hatching. Embryonic changes over the final 10 days of pre‐hatching development have also been documented and proposed as useful staging criteria. However, the reliability of these changes—which focus on the size of the bill and middle toes—as useful staging criteria across different waterfowl breeds has not been fully examined. To evaluate the reliability of these criteria for staging near‐hatching embryos, we examined 27 embryos of Mallard (*Anas platyrhynchos*) and Swan Goose (*Anser cygnoides*). Comparisons with previously published data revealed that size variation within the same developmental stage across breeds is equivalent to within‐breed variation across different stages, suggesting limited reliability of bill and middle toe size for staging waterfowl embryos. Consequently, we devised novel staging criteria for waterfowl based on four easily measurable morphological traits and show that these criteria allow correct stage identification with over 70% accuracy. Our results highlight the importance of quantifying staging accuracy for improving the reliability of embryonic staging tables.

## Graphical abstract

We devised novel staging criteria for near‐hatching embryos of the Mallard (*Anas platyrhynchos*) and Swan Goose (*Anser cygnoides*) based on four easily measurable morphological traits. We show that our staging criteria are robust against interbreed variation and allow for correct stage identification with over 70% accuracy. Our study highlights the importance of assessing staging accuracy, and the reliability of morphological, rather than metric, traits for staging near hatching avian embryos.

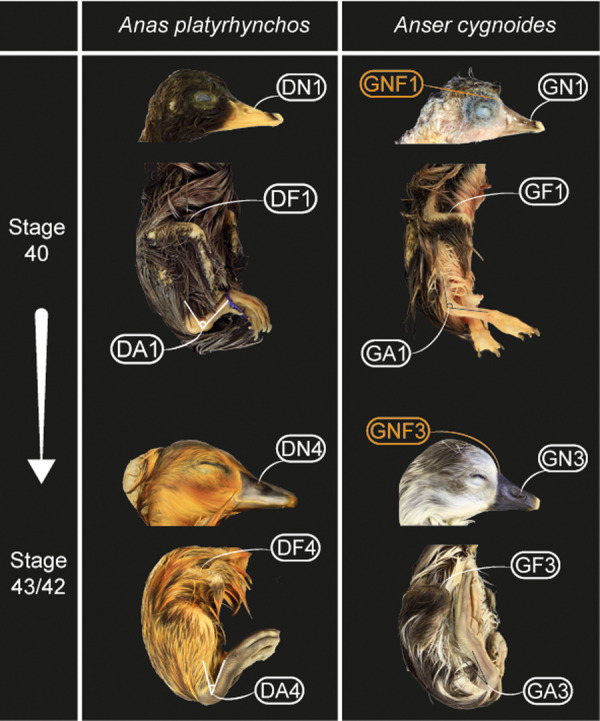

## 1 Introduction

Anseriformes (Waterfowl) is one of the most iconic and economically important groups of birds, factors that have made them the focus of a wide variety of zoological studies, including investigations of embryonic development. Embryological studies of waterfowl have examined the development of many regions of the body including the bill (Brugmann et al. 2010), feet (Zou and Niswander 1996), feathers (Xu, Wu, and Xu 2007), skeleton (Maxwell 2008), liver (Fáncsi 1982), and ovaries (Ran et al. 2023). Moreover, ecological and conservation studies have studied waterfowl embryos for understanding nest parasitism and embryotoxicity of common pollutants (Brunström 1988; Hoffman 1978; Hoffman and Albers 1984; Johnson, Rohwer, and Carloss 1996). Veterinary studies have also examined waterfowl embryos in investigations regarding the viability of artificial insemination (Stunden et al. 1998) and pathology of avian viruses (Bernáth et al. 2006; Fu et al. 2012).

The wide‐ranging relevance of waterfowl embryos across different scientific domains places a premium on establishing reliable and precise normal staging tables. A normal staging table is a description of a species' typical sequence of embryonic features that appear and change between fertilisation and hatching (Stern 2018). Staging tables allow investigators to determine how and when embryonic features and body parts develop. Several waterfowl staging tables have been proposed, most of which are partial (i.e., only covering a portion of development between fertilisation and hatching) and focused only on the early stages of development (Chen 1932; Dupuy et al. 2002, Lukaszewicz et al. 2017). For Mallards (*Anas platyrhynchos*), Chen (1932) described embryonic development up to 12 h post‐fertilisation, and Dupuy et al. (2002) constructed a partial staging table that extends to 72 h post‐fertilisation. For the Swan Goose (*Anser cygnoides*), Lukaszewicz et al. (2017) constructed a partial staging table that extends to 16 h post‐fertilisation.

A few complete staging tables have been devised for the Wood Duck (*Aix sponsa*; Montgomery, Burke, and Byers 1978), ‘giant’ Canada Goose (*Branta canadensis maxima*; Cooper and Batt 1972), and for different breeds of Mallards (Koecke 1958; Li et al. 2019). Koecke (1958) established a staging table using embryos from the Khaki Campbell and Indian Runner breeds, while Li et al. (2019) investigated embryos belonging to the Jinding breed. Li et al. (2019) also constructed a complete staging table for Swan Goose using embryos of the Huo Yan breed. These staging tables applied distinct morphological features for defining embryonic stages 1–39; however, they relied exclusively on measurements of bill and middle toe sizes for the final five stages of development (stages 40–45).

The reliance on organ size for staging embryos may reduce the utility of staging tables, as coeval embryos of different breeds may exhibit different bill and toe sizes (Koecke 1958; Li et al. 2019), a limitation recognised by Ricklefs and Starck (1998). Therefore, establishing broadly applicable staging tables that overcome breed‐specific limitations is necessary for examining near‐hatching embryonic development in a broader range of waterfowl taxa. A more expansive staging table would further facilitate investigations of developmental differences among altricial and precocial species (Ducatez and Field 2021), differences that tend to manifest at later developmental stages (Ricklefs & Starck 1998). To overcome these limitations and benefit future investigations of waterfowl development, the present study proposes novel waterfowl staging criteria based on four morphological traits for near‐hatching Mallard and Swan Goose embryos.

## 2 Materials and Methods

### 2.1 Specimen Acquisition

Sixteen Mallard (*Anas platyrhynchos* Linneus, 1758) eggs and 11 Swan Goose (*Anser cygnoides* Linneus, 1758) eggs were obtained from Anglia Waterfowl and Poultry, Ipswich, UK. Among the Mallards, two were known to belong to the Campbell breed and two to the Runner breed, while the remaining 12 belong to either the Campbell or Runner breeds. The eggs were incubated in a Brinsea Ovation 56 incubator set at 37.5°C and 40% humidity. Mallard eggs were incubated for 18–26 days, and goose eggs for 20–29 days, to obtain embryos older than stage 39 following Li et al. (2019). Mallard embryos were sampled nine times and goose embryos were sampled six times, with one to three replicates at each sampling point (Supporting Information S2: Tables 1 and 2). Embryos were euthanised and extracted from the eggs following UK Home Office Regulatory guidelines, fixed in Paraformaldehyde (PFA) overnight, then dehydrated using an ethanol series the next day. Ages of the extracted embryos and their replicates are listed in Supporting Information S2: Tables 1 and 2. Additionally, two deceased *A. platyrhynchos* embryos and 14 *A. cygnoides* embryos of unknown age were obtained from the same source and were used to assess the precision of our proposed staging criteria (Supporting Information S2: Tables 1 and 2).

### 2.2 Size Measurements

Bill and middle toe lengths were measured using digital callipers accurate to 0.2 mm and compared with previously published measurements (Koecke 1958; Li et al. 2019) to assess the reliability of the use of size measurements for staging duck and goose embryos.

### 2.3 Morphological Criteria for Staging Embryos

To establish morphological criteria for staging embryos of each species, detailed observations were made using a light stereomicroscope. Specifically, we focused on observations of the development of the nostril, nasofrontal hinge, ankles and wing feather tracts. We measured the angle of ankle flexure, that is, the angle formed between the tibiotarsus and tarsometatarsus, by manually pulling together the medial sides of the right and left feet of the embryo. Afterwards, we aligned the arms of a compass parallel to the left tibiotarsus and tarsometatarsus, then measured the angle of the compass with a protractor. Changes in the transparency of the foot webbing, as described by Caldwell and Snart (1974), were observed by manual separation of the toes and looking through the webbing. Afterwards, the development of these traits was divided into phases.

We constructed the staging criteria for each species by tabulating the age of each embryo and the phase of development of each trait. Embryos of similar ages and phases of trait development were grouped together. After grouping the embryos, the mean and/or modal phase of trait development was calculated for each group, and a stage number was assigned to each group with their corresponding age range and phases of trait development.

### 2.4 Precision of Staging Criteria

The precision of our staging criteria, assessed as interobserver variability, was determined by independent staging of several randomly selected embryos by six independent researchers, three for each species. Before each trial, embryos were placed in containers covered in foil, then shuffled in their placement to ensure randomness of the embryo chosen for staging. Subsequently, during the trial, an embryo was picked at random and the phase of development of each trait within the embryo was determined and tabulated. At the end of each trial, the developmental phases of each trait for each embryo were used to estimate its embryonic stage, or range of stages, based on the staging criteria. At the end of all the trials, the estimated stages for each embryo were compiled and compared. The trials for each species were analysed separately.

Deviations among estimated stages for each embryo were quantified by number and magnitude. The magnitude of deviation was assigned a value of 0.5 when two staging estimates partially overlapped. Estimates deviating by one stage were assigned a value of 1, estimates deviating by two stages were assigned a value of 2, and so on. Afterwards, we multiplied the total number of deviations with their respective magnitude to obtain a weighted assessment of deviation for each embryo, and this weighted total was divided by the total number of staging trials and converted to a percentage, dubbed the ‘deviation score’. Finally, the compliment of the deviation score, dubbed the ‘similarity score’, was calculated.

### 2.5 Specimen Imaging

Photographs of the nostrils, nasofrontal hinges, and wing feather tracts were taken at the University of Cambridge Museum of Zoology (UMZC). Photographs were taken using a Canon DSLR with a 1.6x lens mounted on a Leica Z16 APO zoom system. The anterior alar tract of a goose embryo at stage 41 was stained with blue ink (Parker QUINK) to provide contrast. Photographs of the embryos and ankles were taken with a 100 mm lens mounted on a vertical stand. The photographs were taken at multiple focal planes and stacked into images using Helicon Focus (7.5.6 Pro). Image backgrounds were cropped using Adobe Photoshop 23.3.2 20220503.

## 3 Results

### 3.1 Bill and Middle Toe Size Variation

Mean bill and middle toe lengths increased with age in our sample, with little variance within each age group in both species (Tables 1 and 2). However, comparisons of our measurements to other studies (Koecke 1958; Li et al. 2019), which used the black khaki Campbell and Jinding Mallard breeds and the Huo Yan goose breed, revealed a great deal of intraspecific variation (Table 1 and 2). Indeed, size differences among coeval embryos from different breeds approximate or even exceed size differences observed among different age groups within a breed (Tables 1 and 2).

**Table 1:** Comparison of beak and middle toe lengths for 18–26 day‐old embryos from different breeds of the Mallard duck *Anas platyrhynchos*.

		This study		Koecke (1958)^a^	Li et al. (2019)^b^	
		Campbell and Runner breeds		Black Khaki Campbell breed	Jinding breed	
Day	Replicate no.	BL (mm)	TL (mm)	BL (mm)	BL (mm)	TL (mm)
18	3	11.8 ± 1.1	16 ± 4.7	13	14.7	16.9
19–20	3	11.7 ± 0.8	18.8 ± 2.8	—	15.2	19.3
21–22	3	12.5 ± 0.45	21 ± 2.3	15	15.4	19.7
23–24	2	12.6 ± 1.7	20.7 ± 3.5	17	16.5	20.6
25–26	5	13.36 ± 0.7	24.4 ± 2.3	—	17.3	22.7

Abbreviations: BL = beak length, TL = middle toe length.

a Taken from Koecke (1958).

^b^ Taken from Li et al. (2019).

**Table 2:** Comparison of beak and middle toe lengths of 19–29 day‐old embryos of the domesticated Swan Goose *Anser cygnoides*.

Day	Replicate no.	This study Chinese breed		Li et al. (2019)^a^ Huo Yan breed	
		BL (mm)	TL (mm)	BL (mm)	TL (mm)
19	2	15 ± 1.4	14 ± 1.4	13.1	14.5
20	2	16 ± 1.4	17.5 ± 0.7	14.2	16.8
21	2	17 ± 1.4	19.5 ± 0.7	14.9	18.3
24	2	18 ± 0.0	28 ± 2.8	15.5	20.4
26	2	19.5 ± 0.7	27 ± 1.4	16.3	22.7
29	1	19	26	—	—

Abbreviations: BL = beak length, TL = middle toe length.

^a^ Taken from Li et al. (2019).

### 3.2 Discrete Morphological Changes

Seven easily observable morphological traits were found to change over time in a broadly consistent manner in waterfowl embryos, suggesting that they may be valuable in the establishment of a consistent staging criteria. These are described below.

### 3.3 Nostril Development in Mallard

The development of the nostril and the visible components of the nasal cavity are described in lateral view and are divided into four phases, denoted with the abbreviation ‘DN’.

#### 3.3.1 Dn1

**Figure 1:**
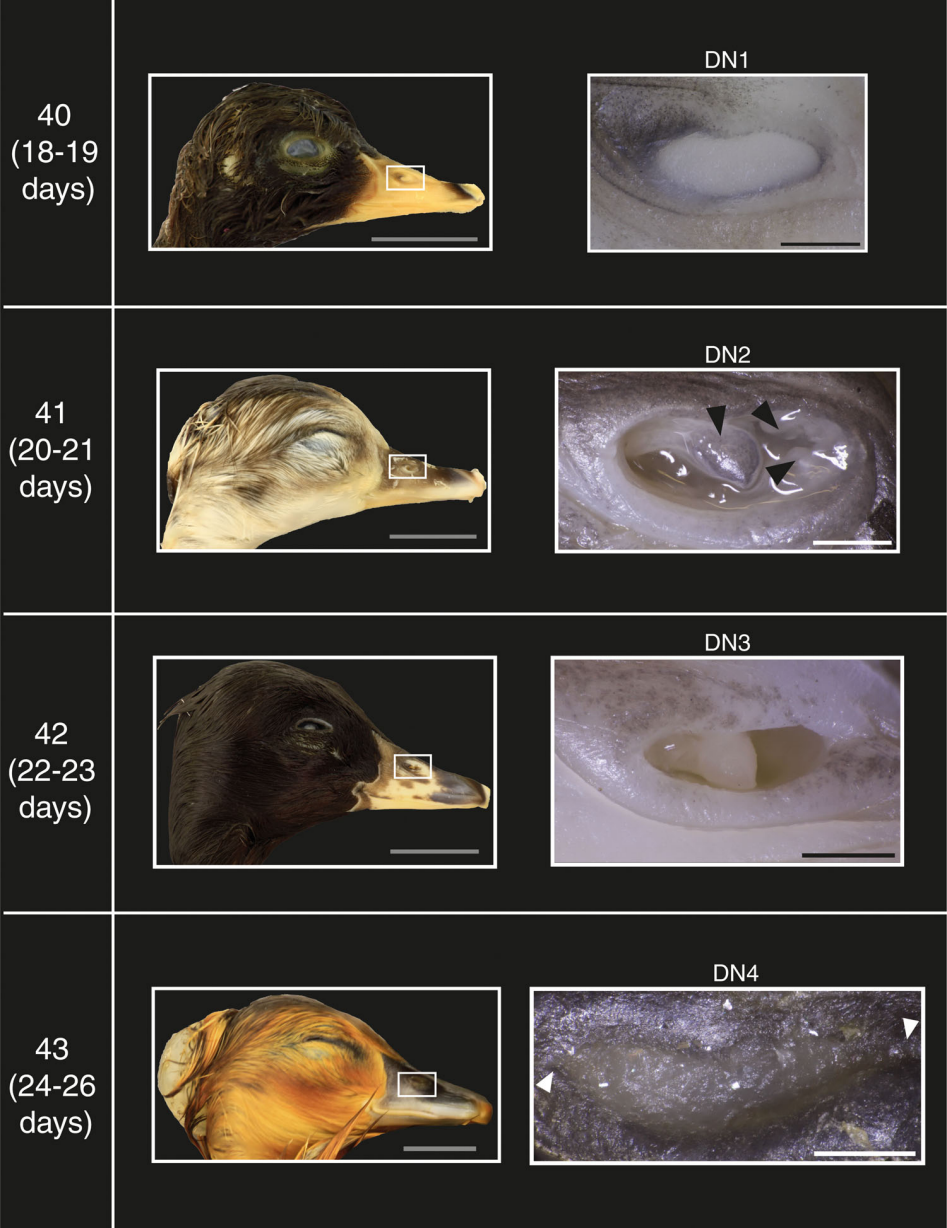
Part of the staging criteria for near‐hatching embryos of the Mallard duck (*Anas platyrhynchos*) illustrating nostril external development. DN (1–4): Phases of nostril development. DN2: In the second phase of nostril development four nasal components appear, indicated by black arrowheads. DN4: The narrow rostral and caudal ends are indicated by white arrowheads. Grey scale bars are 1 cm and other scale bars are 1 mm.

After 18–19 days of incubation, the nostrils of most embryos appear rostrocaudally elongate with dorsoventrally deep rostral and caudal ends, yielding a peanut‐like shape. The cavity lacks any visible nasal components, but has a blank white surface (Figure 1, Stage 40 DN1).

#### 3.3.2 DN2

After 20–21 days of incubation, the overall shape of the nostril appears similar to the previous stage. However, four nostril components appear at this stage: a large triangular caudolaterally directed component that appears near the caudal end of the cavity, and three triangular rostral components that are directed caudally (Figure 1, Stage 41 DN2).

#### 3.3.3 DN3

After 23–24 days of incubation, the nostril of most embryos is rostrocaudally elongate and is narrower dorsoventrally than in previous stages. Moreover, the three rostral triangular components are no longer visible and only the large caudal component remains visible (Figure 1, Stage 42 DN3).

#### 3.3.4 DN4

After 23–24 days of incubation, the nostril of most embryos is elongated relative to previous stages and takes the form of a slit with pointed rostral and caudal ends (Figure 1, Stage 43 DN4).

### 3.4 Nostril Development in Geese

The development of the nostril and its position within the bill is described in lateral view and is divided into four phases, denoted with the abbreviation ‘GN’.

#### 3.4.1 GN1

**Figure 2:**
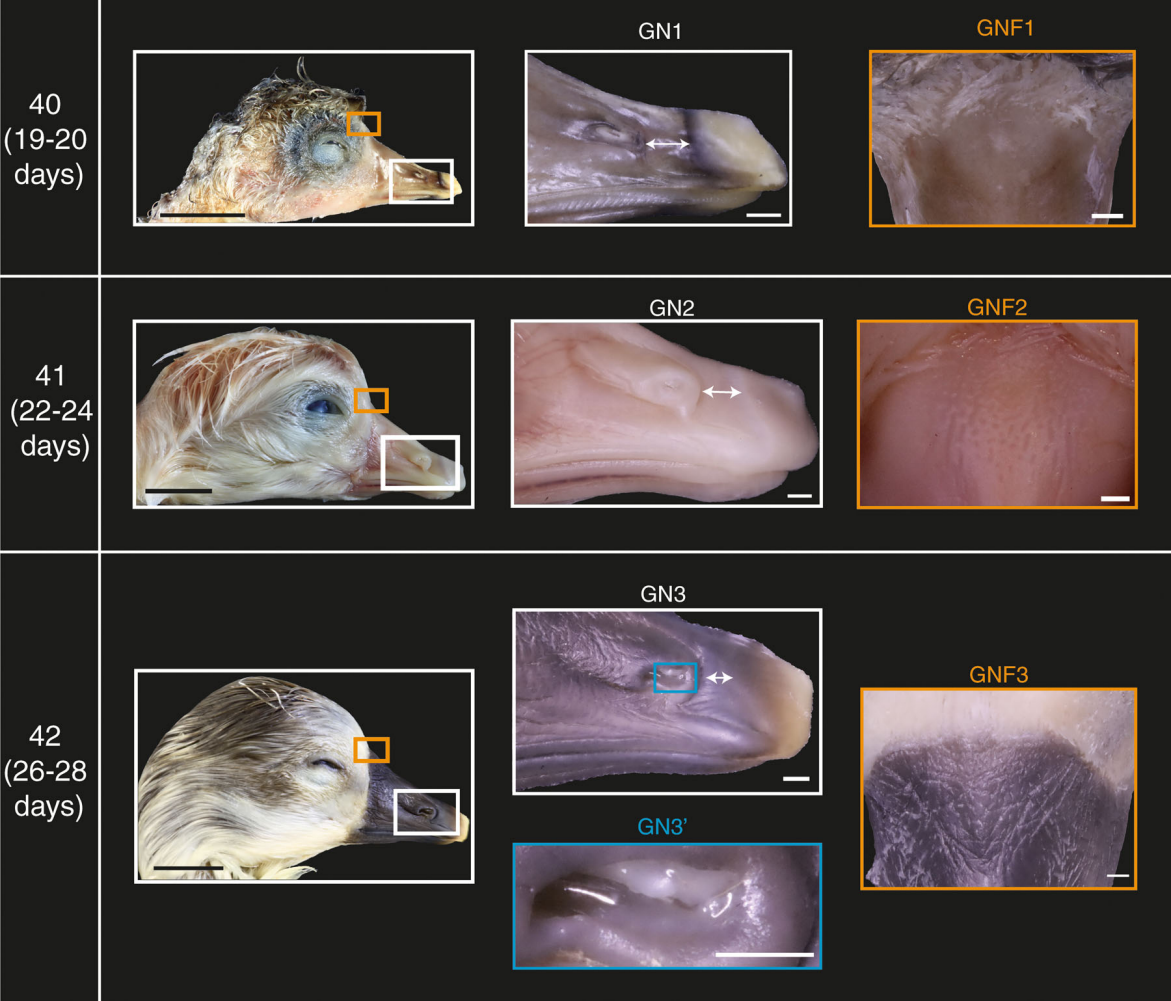
Part of the staging criteria for near‐hatching embryos of Swan Goose (*Anser cygnoides*) illustrating nostril and beak external development. GN (1–3): Phases of nostril development. In the first and second phases the distance between the nostril and the egg tooth is indicated with a double‐headed arrow (GN1‐2). GN3: At the third phase a pointed white nasal component appears. GNF (1–3): Phases of nasofrontal hinge development. Black scale bars are 1 cm and white scale bars are 1 mm.

After 19–20 days of incubation the nostril is prominent, and its rostral extremity is located approximately 1 mm caudal to the back of the egg tooth. The nasal cavity lacks any visible nasal components but is oval in shape and exhibits a blank white surface (Figure 2, GN1).

#### 3.4.2 GN2

After 20–24 days of incubation, the nostril of most embryos extends rostrally such that it is situated closer to the caudal end of the egg tooth than the previous phase. Moreover, the nostril cavity begins to increase in depth on its rostral side, which gives the nostril a roughly triangular appearance (Figure 2, GN2). In some embryos, a small light yellow cartilaginous process extends from inside the nasal cavity to the dorsorostral edge of the nostril.

#### 3.4.3 GN3

After 24–29 days of incubation, the nostril of most embryos appears to contact the caudal end of the egg tooth. The nostril cavity acquires a dorsoventrally deeper rostral end and exhibits a more prominent internal cartilaginous process than the previous phase (Figure 2, GN3 & GN3′).

### 3.5 Ankle Flexure Development in Mallards and Geese

The angle of ankle flexure is measured and described in lateral view. Overall, this angle decreases as development progresses in both species. This process is divided into three phases, denoted as ‘DA’ for mallards and ‘GA’ for geese.

#### 3.5.1 DA1 and GA1

**Figure 3:**
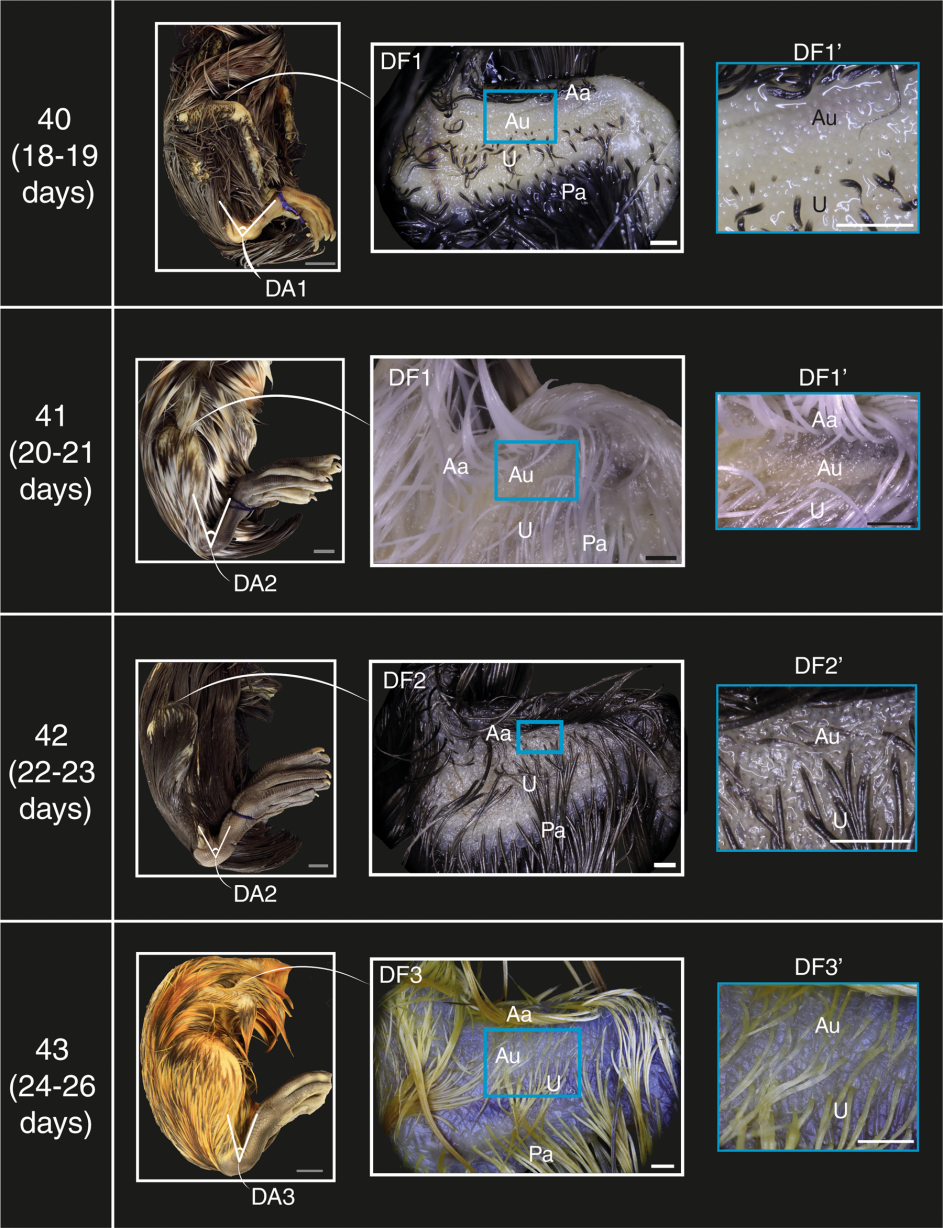
Part of the staging criteria for near‐hatching embryos of the Mallard duck (*Anas platyrhynchos*) illustrating postcranial development. DA (1–3): phases of changes in angle of ankle flexure. DF (1–3): Phases of feather tract development. DF1': inset showing the absence of anterior ulnoradial tract at stages 40 and 41. DF2': inset showing a short anterior ulnoradial tract at stage 42. DF3': inset showing a longer anterior ulnoradial tract at stage 43. DA (1–3): Phases of ankle development. Grey scale bars are 1 cm and other scale bars are 1 mm. Aa; anterior alar, Au; anterior ulnoradial, Pa; posterior alar tracts; U; ulnoradial.

**Figure 4:**
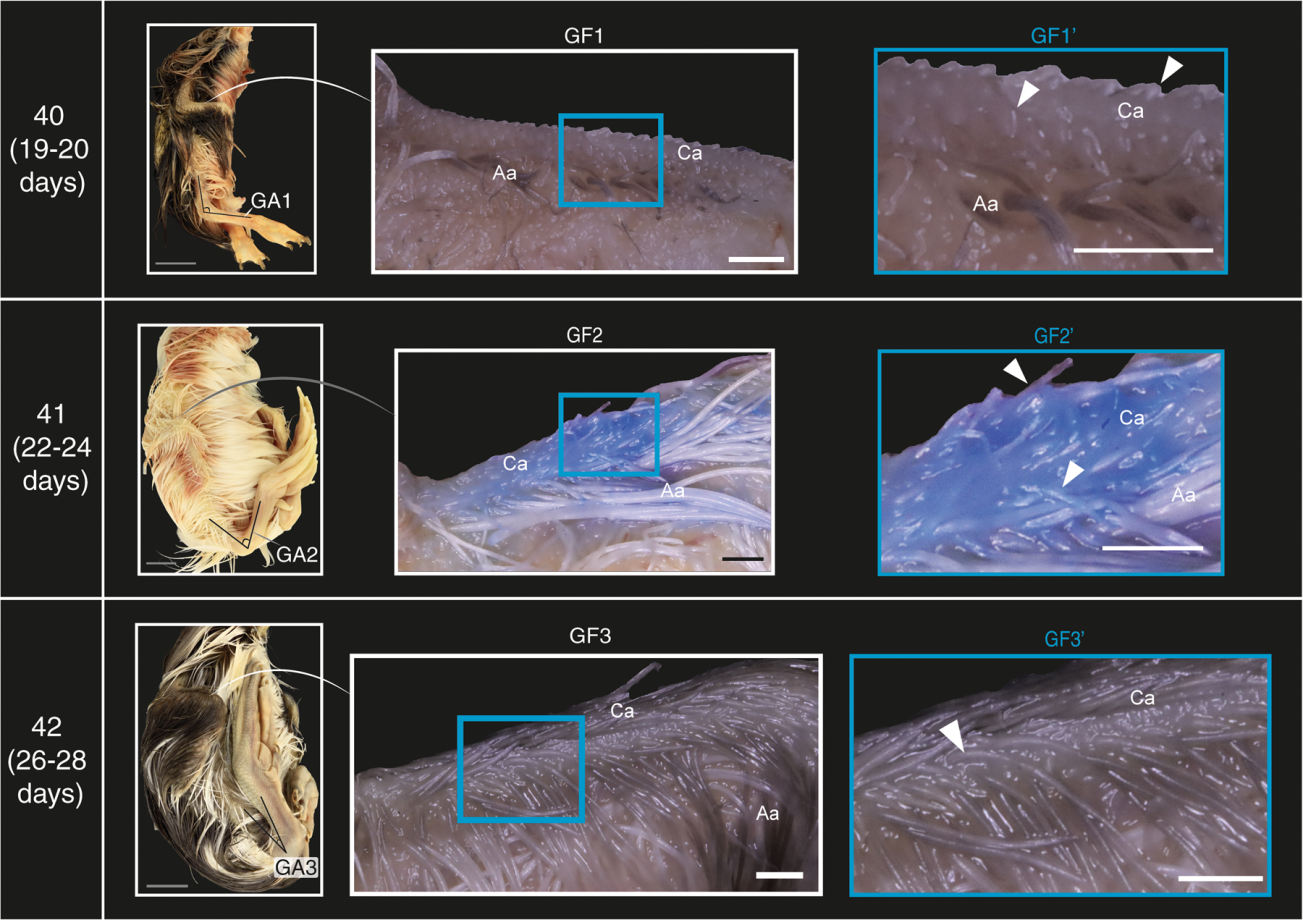
Part of the staging criteria for near‐hatching embryos of the Swan Goose (*Anser cygnoides*) illustrating postcranial development. GF (1–3): Phases of cranial feather tract development; tracts are indicated with black arrowheads. GA (1–3): Phases of ankle development. Grey scale bars are 1 cm and other scale bars are 1 mm. Aa; anterior alar, Ca; cranial alar.

After 18–19 days of incubation, the angle of ankle flexure in most mallards reaches its greatest value (i.e., most obtuse angle), with a mean angle of ~46° (Figure 3, Stage 40 DA1; Supporting Information S2: Table 1). In geese, after 19–20 days of incubation embryos exhibit a mean angle of 81° (Figure 4, Stage 40 GA1; Supporting Information S2: Table 2).

#### 3.5.2 DA2 and GA2

The angle of ankle flexure in most mallards after 20–24 days of incubation is smaller than the previous stage, with a mean angle of ~27° (Figure 3, Stages 41 and 42 DA2; Supporting Information S2: Table 1). In geese, after 22–24 days of incubation embryos exhibit a mean angle of 42 (Figure 4, Stage 41 GA2; Supporting Information S2: Table 2).

#### 3.5.3 DA3 and GA3

The angle of ankle flexure in most mallards after 20–24 days of incubation is smallest at this stage, with a mean angle of 5° (Figure 3, Stage 43 DA3; Supporting Information S2: Table 1). As for geese, most embryos after 22–24 days of incubation have a mean angle of 15° (Figure 4, Stage 42 GA3; Supporting Information S2: Table 2).

### 3.6 Underwing Feather Tract Development in Mallards

The underwing feather tracts were labelled along the cranial‐caudal axis of the wing as the anterior alar, anterior ulnoradial, ulnoradial and posterior alar tracts, based on Gill (2007).

#### 3.6.1 DF1

**Figure 5:**
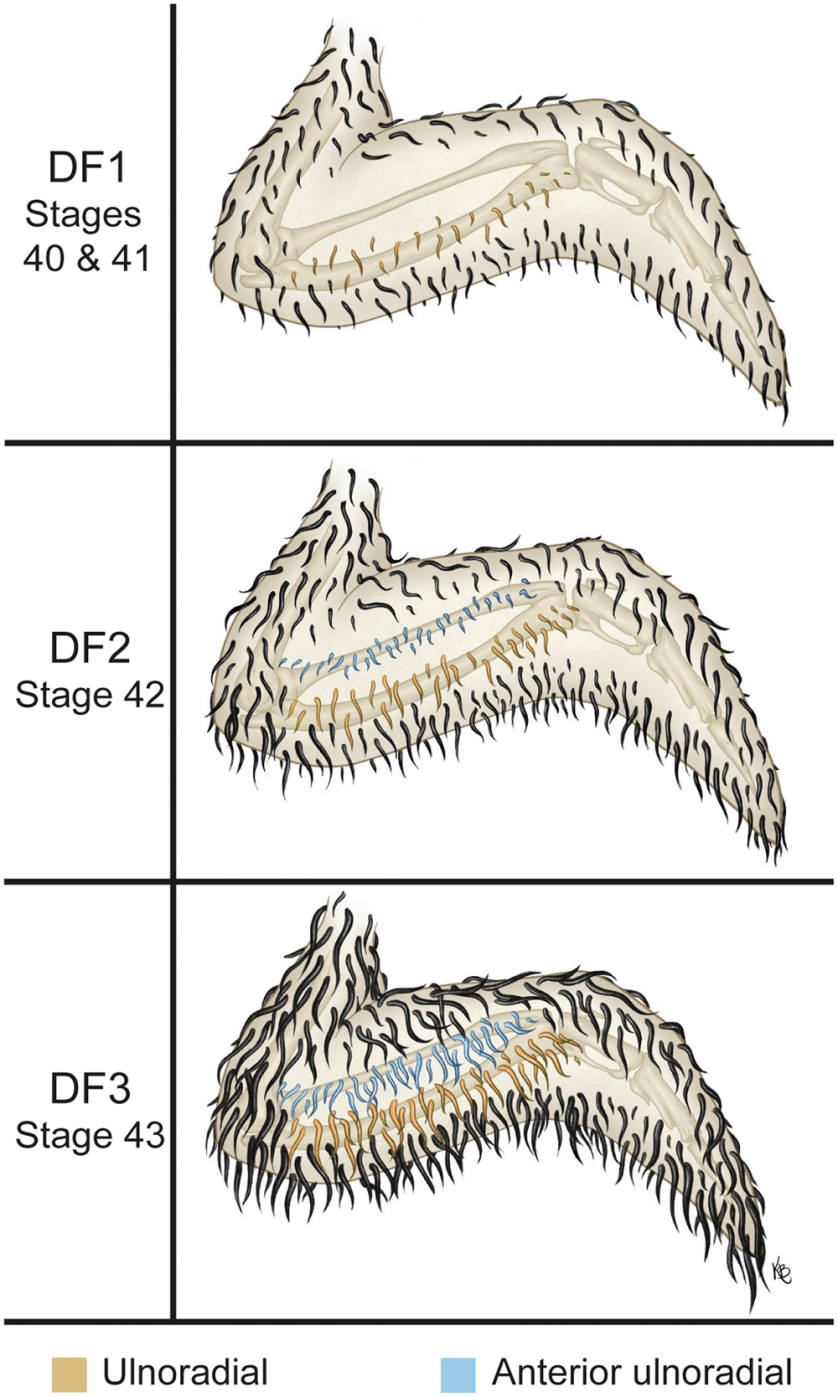
Stages of wing pterylae development in near‐hatching Mallard duck (*Anas platyrhynchos*) embryos used in our staging criteria.

After 18–19 days of incubation most embryos had developed the anterior alar, ulnoradial and posterior alar tracts. The ulnoradial tract has the smallest feather primordia, and the anterior ulnoradial tract has not yet formed (Figures 3 and 5, Stages 40 and 41 DF1, DF1′).

#### 3.6.2 DF2

After 20–23 days of incubation most embryos exhibit elongate feather primordia of the ulnoradial tract that overlap the posterior alar tract caudally. The anterior ulnoradial tract appears between the anterior alar and ulnoradial tract as small feather primordia that do not extend into the ulnoradial tract (Figures 3 and 5, Stage 42 DF2, DF2′).

#### 3.6.3 DF3

After 20–26 days of incubation most embryos exhibit an elongated primordia of anterior ulnoradial tract overlapping the ulnoradial tract (Figures 3 and 5, Stage 43 DF3, DF3′).

### 3.7 Cranial Wing Feather Tract Development in Geese

The development of the alar feather tract on the cranial side of the wing was divided into three phases, denoted with the abbreviation ‘GF’.

#### 3.7.1 GF1

**Figure 6:**
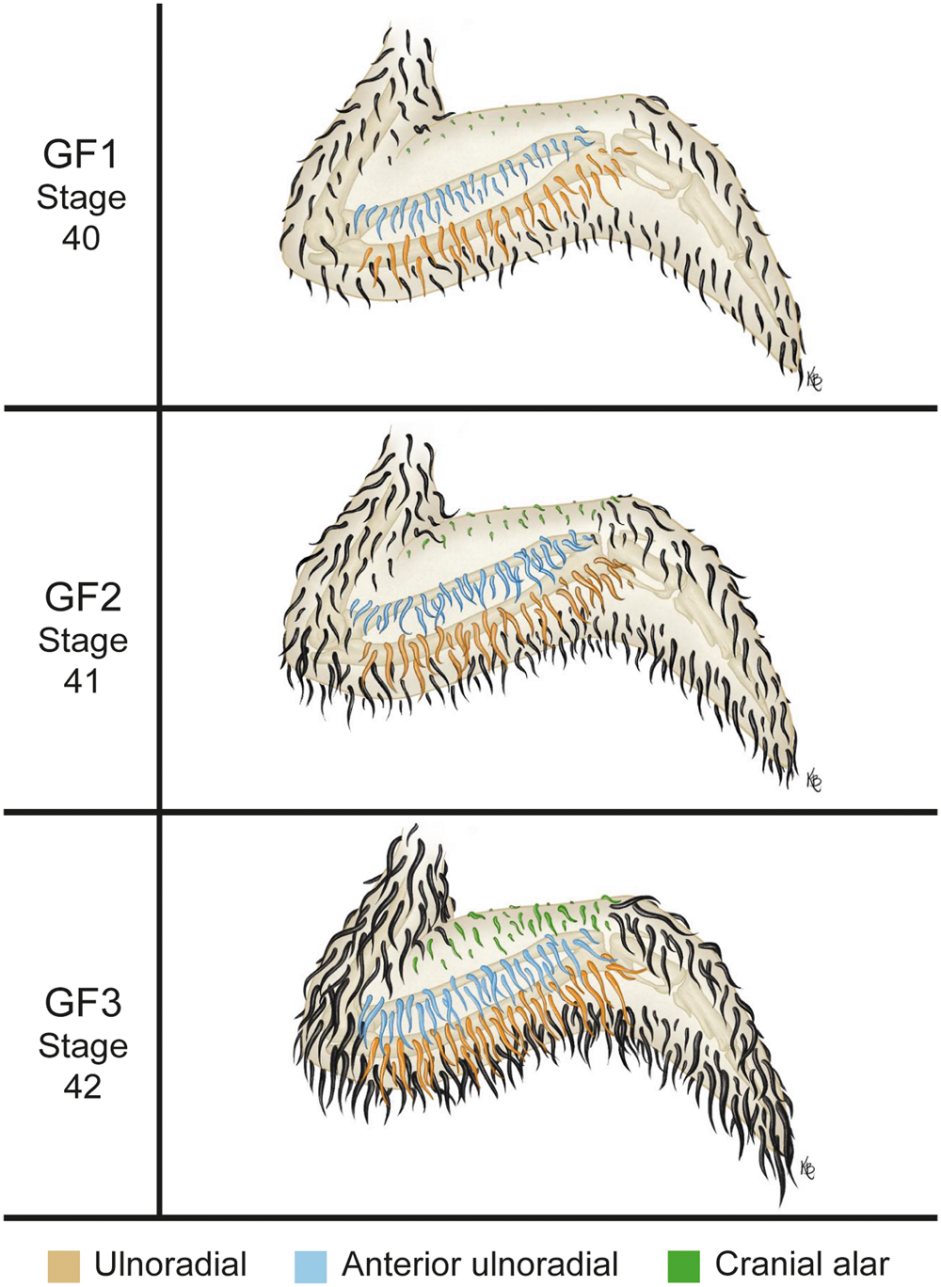
Stages of wing pterylae development in near‐hatching Swan Goose (*Anser cygnoides*) embryos used in the staging criteria.

After 19–20 days of incubation most goose embryos exhibit small feather papilla on the cranial side of the proximal humeral tract (Figures 4 and 6, Stage 40 GF1, GF1′).

#### 3.7.2 GF2

After 20–24 days of incubation most goose embryos exhibit very short feather follicles on the cranial side of the proximal humeral tract (Figures 4 and 6, Stage 41 GF2, GF2′).

#### 3.7.3 GF3

After 26–29 days of incubation most goose embryos have longer feather follicles on the cranial side of the proximal humeral tract relative to the previous stage, (Figures 4 and 6, Stage 42 GF3, GF3′).

### 3.8 Transparency of Foot Webbing in Mallards

Changes in the transparency of the webbing between the toes were divided into three phases, denoted with the abbreviation ‘DT’.

#### 3.8.1 DT1

The webbing between the toes of most embryos after 18–19 days of incubation is transparent, such that the shape and colour of objects can be discerned when looking through the webbing.

#### 3.8.2 DT2

The webbing between the toes of most embryos after 20–26 days of incubation is less transparent so that only the colours of objects are discernible when looking through the webbing.

#### 3.8.3 DT3

The webbing between the toes of most embryos after 24–26 days of incubation is opaque such that neither the colour nor shape of objects on either side of it are discernible.

### 3.9 Development of the Frontonasal Hinge in Geese

The rostral half of the frontonasal hinge is at the caudodorsal end of the beak and is composed of keratin, lacking any feathers. The development of this region of the hinge was divided into three phases, denoted with the abbreviation ‘GNF’.

#### 3.9.1 GNF1

After 19–20 days of incubation most goose embryos exhibit a smooth, featureless curved surface at the frontonasal hinge (Figure 2, Stage 40 GNF1).

#### 3.9.2 GNF2

The frontonasal hinges of most goose embryos after 22–24 days of incubation have deep pits that lack any specific arrangement (Figure 2, Stage 41 GNF2).

#### 3.9.3 GNF3

Most goose embryos after 26–29 days of incubation have deep, elongated pits that form a triangular ridge at the frontonasal hinge (Figure 2, Stage 42 GNF3).

### 3.10 Staging Criteria

The phase of development of each trait in each embryo was tabulated, along with the mean and modal phase for each group (Supporting Information S2: Tables S1 and S2). The devised staging criteria consist of four stages for mallards and three for geese (Figures 1, 2, 3, 4, 5, 6, Supporting Information S1: Figures S1 and S2 and Tables 3 and 4). The precision of our staging criteria was assessed with staging trials (Supporting Information Table S3). The results of the trials revealed a similarity score for the mallard staging criterion of 77% and 74% for the goose staging criterion (Supporting Information: Table S4). Furthermore, the staging of Mallard embryos was mostly consistent across embryonic stages. As for goose embryos, the consistency was lower than Mallard embryos, with stage 41 being the most inconsistent. However, most of the staging inconsistency occurred when staging deceased goose embryos.

**Table 3:** Criteria for staging near‐hatching embryos of the Mallard duck (*Anas platyrhynchos*) based on four morphological traits.

Stage	Age (no. of incubation days)	DA	DF	DN	DT
40	18–19	1	1	1	1
41	20–21	2	2	2	2
42	22–23			3	
43	24–26	3	3	4	2–3

Abbreviations: A, angle of ankle flexure; F, feather tract; N, nostril; T, transparency of foot webbing.

**Table 4:** Criteria for staging near‐hatching embryos of the domesticated Swan Goose (*Anser cygnoides*) based on four morphological traits.

Stage	Age (no. of incubation days)	GN	GA	GF	GNF
40	19–20	1	1	1	1
41	22–24	2	2	2–3	2
42	26–28	3	3	3	3

Abbreviations: A, angle of ankle flexure; F, feather tract; N, nostril; NF, nasofrontal hinge.

## 4 Discussion

Despite the popularity of the use of bill and middle toe sizes in staging near‐hatching avian embryos such as chickens (Hamburger and Hamilton 1992), Japanese Quail (Ainsworth, Stanley, and Evans 2010), ducks and geese (Li et al. 2019), these criteria appear to be of limited utility when different or unknown breeds are used. This limitation was apparent from comparison of the lengths of the bill and middle‐toes of duck and goose embryos of the same age range from different breeds (Tables 1 and 2). These comparisons revealed that the interbreed variance in bill and middle toe lengths of coeval embryos is similar to the intrabreed variance in bill or middle toe lengths between embryos of different ages (Tables 1 and 2). The equivalence of these variances indicates limited reliability of bill and middle toe lengths in staging waterfowl embryos.

The limitations of using size for staging near‐hatching embryos illustrate the need for a more robust alternative morphology‐based staging table. Some of the morphological traits that we used have been applied as staging criteria for other bird taxa; for instance, the angle of ankle flexure in embryos of the Society Finch (*Lonchura striata var. domestica*; (Yamasaki and Tonosaki 1988)). Caldwell and Snart (1974) used the transparency of foot webbing for indexing duck embryos, although they did not establish a staging table. The major difficulty of establishing a high‐precision staging table is the considerable degree of intraspecific variation in the rate of development of different morphological traits. To overcome this challenge, we devised a staging criterion that indicates the morphological features that are most likely to be observed for embryos within a particular age range. Consequently, our criteria have three advantages over previously published staging tables for these species. First, our criteria should be applicable to different breeds of ducks, as they were devised using morphological features that commonly occur in two different duck breeds. These morphological features were not discussed in the recent staging table proposed by Li et al. (2019). The use of four morphological features is the second advantage of our staging criteria: Koecke (1958) only mentioned two morphological features rather than the four applied in our criteria. The variability in developmental rate of any individual trait illustrates the benefit of a staging scheme involving multiple traits: the most likely stage for each embryo remains discernable based on the development of all traits considered collectively. The third advantage is the high precision of staging criteria, which ensures the production of consistant staging by different investigators. Consistency in staging embryos is crucial to prevent assigning different stages to isochronic developmental features and events by different investigators. However, precision of staging tables and staging criteria are not commonly reported in the published literature.

### 4.1 Reliability of Staging Criteria

In devising useful staging criteria, accuracy, precision and robustness should be evaluated. Accurate criteria enable embryos of different stages to be distinguished with little ambiquity, while precise criteria involve little interobserver variability, ensuring that different investigators assign the same stage to embryos exhibiting the same features. Robustness is the extent to which criteria are recognizable despite the variation present among embryos of different breeds, populations or genetic backgrounds. Ideally, these factors should be quantified for proposed staging criteria, with statistical methods that account for necessary sample sizes, and unified with a single score, though this is rarely done. Our results indicate that previously published staging tables for Anseriformes are not robust to interbreed variance, highlighting the need to devise new staging criteria for this group. As for precision, we devised a method for quantifying precision, similarity score, which would indicate the extent of interobserver variability. The adaquency of the similarity score, or indeed any other measure of precision, requires detailed analysis with larger sample sizes.

### 4.2 Implications for Comparative Embryological Studies

We attempted to develop comparative staging criteria that are applicable for multiple species of anseriforms, as previously attempted by Li et al. (2019). Constructing comparative staging criteria depends on the appearance of similar features in different species. Problematically, at near‐hatching stages, we could not identify any easily observable external morphological traits that are common to both species investigated. The absence of common developmental traits at near‐hatching stages may suggest an increase in whole‐embryo interspecific variability during external embryonic development. This finding is consistent with the observation that interspecific variablity in growth duration, which may be correlated with phenotypic disparity, is highest at HH stage 33 and above (Cooney et al. 2020).

The occurance of common developmental traits between Mallard and goose embryos from stages 1 to 39, and the subsequent disappearance of any common features at later stages has several important implications. One implication is that HH stage 40 might approximate the point of embryonic divergence in external morphology between precocial bird species. This divergence point is close that previously documented between precocial and altricial bird species, at HH stage 39 (Ricklefs and Starck 1998), implying that avian external embryonic development is more constrained at earlier embryonic stages than at near‐hatching stages, and that staging criteria are clearly applicable across species only at comparatively early embryonic stages. This implication is further supported by the lack of easily identifiable interspecific features appearing at these later stages, as well as the sequence heterogenity in wing pterylae formation and apparent heterochrony in the rate of change of ankle flexure. The plasticity of embryonic development at these late‐stages indicates that external morphology alone might be of limited utility for constructing broadly applicable staging criteria across multiple species. Furthermore, it is indicative of the necessity of assessing internal features for comparative multispecies staging studies, as exemplified by Ricklefs and Starck (1998).

### 4.3 Applications of the Staging Criteria

Our staging criteria can be used for examining duck and goose embryological development at stages close to hatching, which will be beneficial for the exploration of parameters such as osteocranium and chondrocranium development (Maxwell 2008), feather tract development, foot scale cornification (Koecke 1958), foot webbing pigmentation, wing pterylae and tongues (Louryan et al. 2023), liver (Bao et al. 2023), gonads (Mizia et al. 2023), and muscle formation (Guo et al. 2021). We hope our approach to staging these taxa will help facilitate a range of investigations into anseriform development in the coming years. We further hope that assessing staging precision will become common practice in devising new embryonic staging tables.

## Author Contributions

**Bassel Arnaout:** conceptualization, data curation, formal analysis, methodology, writing – original draft, scientific illustrations, writing – review and editing. **Kaylen Brzezinski:** providing scientific illustrations. **Benjamin Steventon:** conceptualization, formal analysis, funding acquisition, project administration, resources, supervision, writing – review and editing. **Daniel J. Field:** conceptualization, formal analysis, funding acquisition, project administration, resources, supervision, writing – review and editing.

## Conflicts of Interest

The authors declare no conflicts of interest.

## Supporting information

Supporting information.

Supporting information.

## Data Availability

The data that support the findings of this study are available from the corresponding author upon reasonable request.

## References

[jmor70027-bib-0001] Ainsworth, S. J. , R. L. Stanley , and D. J. R. Evans . 2010. “Developmental Stages of the Japanese Quail.” Journal of Anatomy 216, no. 1: 3–15. 10.1111/j.1469-7580.2009.01173.x. 19929907 PMC2807971

[jmor70027-bib-0002] Bao, Q. , L. Wang , X. Hu , et al. 2023. “Developmental Changes of Duckling Liver and Isolation of Primary Hepatocytes.” Animals : An Open Access Journal From MDPI 13, no. 11: 1820. 10.3390/ani13111820. 37889689 PMC10252113

[jmor70027-bib-0003] Bernáth, S. , A. Farsang , A. Kovács , E. Nagy , and M. Dobos‐Kovács . 2006. “Pathology of Goose Haemorrhagic Polyomavirus Infection in Goose Embryos.” Avian Pathology 35, no. 1: 49–52. 10.1080/03079450500465759. 16448943

[jmor70027-bib-0004] Brugmann, S. A. , K. E. Powder , N. M. Young , et al. 2010. “Comparative Gene Expression Analysis of Avian Embryonic Facial Structures Reveals New Candidates for Human Craniofacial Disorders.” Human Molecular Genetics 19, no. 5: 920–930. 10.1093/hmg/ddp559. 20015954 PMC2816616

[jmor70027-bib-0005] Brunström, B. 1988. “Sensitivity of Embryos From Duck, Goose, Herring Gull, and Various Chicken Breeds to 3,3′,4,4′‐Tetrachlorobiphenyl.” Poultry Science 67, no. 1: 52–57. 10.3382/ps.0670052. 3131755

[jmor70027-bib-1001] Caldwell, P. J. , and A. E. Snart . 1974. “A Photographic Index for Aging Mallard Embryos.” Journal of Wildlife Management 38, no. 2: 298–301. 10.2307/3800736.

[jmor70027-bib-0006] Cooney, C. R. , C. Sheard , A. D. Clark , et al. 2020. “Ecology and Allometry Predict the Evolution of Avian Developmental Durations.” Nature Communications 11, no. 1: 2383. 10.1038/s41467-020-16257-x. PMC7224302 32409662

[jmor70027-bib-0007] Cooper, J. A. , and B. D. J. Batt . 1972. “Criteria for Aging Giant Canada Goose Embryos.” Journal of Wildlife Management 36, no. 4: 1267–1270. 10.2307/3799260.

[jmor70027-bib-1003] Chen, B. K. 1932. “The Early Development of the Duck's Egg, With Special Reference to the Origin of the Primitive Streak.” Journal of Morphology 53, no. 1: 133–187. 10.1002/jmor.1050530106.

[jmor70027-bib-0008] Ducatez, S. , and D. J. Field . 2021. “Disentangling the Avian Altricial‐Precocial Spectrum: Quantitative Assessment of Developmental Mode, Phylogenetic Signal, and Dimensionality.” Evolution 75, no. 11: 2717–2735. 10.1111/evo.14365. 34608994

[jmor70027-bib-1004] Dupuy, V. , B. Nersessian , and M. R. Bakst . 2002. “Embryonic Development From First Cleavage Through Seventy‐Two Hours Incubation in Two Strains of Pekin Duck (Anas platyrhynchos.” Poultry Science 81, no. 6: 860–868. 10.1093/ps/81.6.860. 12079054

[jmor70027-bib-0009] Fáncsi, T. 1982. “Ultrastructural Studies of the Goose Embryo Liver.” Anatomia, Histologia, Embryologia: Journal of Veterinary Medicine Series C 11, no. 2: 138–146. 10.1111/j.1439-0264.1982.tb00929.x. 6214969

[jmor70027-bib-0010] Fu, Y. , Z. Chen , C. Li , and G. Liu . 2012. “Establishment of a Duck Cell Line Susceptible to Duck Hepatitis Virus Type 1.” Journal of Virological Methods 184, no. 1–2: 41–45. 10.1016/j.jviromet.2012.05.004. 22633926

[jmor70027-bib-0011] Guo, B. B. , Z. C. Dai , Y. H. Ren , et al. 2021. “Improvement of Goose Embryonic and Muscular Developments by Wider Angle Egg Turning During Incubation and the Regulatory Mechanisms.” Poultry Science 100, no. 12: 101477. 10.1016/j.psj.2021.101477 PMC8554260 34695628

[jmor70027-bib-0012] Hamburger, V. , and H. L. Hamilton . 1992. “A Series of Normal Stages in the Development of the Chick Embryo.” Developmental Dynamics 195, no. 4: 231–272. 10.1002/aja.1001950404. 1304821

[jmor70027-bib-0013] Hoffman, D. J. 1978. “Embryotoxic Effects of Crude Oil in Mallard Ducks and Chicks.” Toxicology and Applied Pharmacology 46, no. 1: 183–190. 10.1016/0041-008x(78)90149-7. 725942

[jmor70027-bib-0014] Hoffman, D. J. , and P. H. Albers . 1984. “Evaluation of Potential Embryotoxicity and Teratogenicity of 42 Herbicides, Insecticides, and Petroleum Contaminants to Mallard Eggs.” Archives of Environmental Contamination and Toxicology 13, no. 1: 15–27. 10.1007/bf01055642. 6703782

[jmor70027-bib-0015] Johnson, W. P. , F. C. Rohwer , and M. Carloss . 1996. “Evidence of Nest Parasitism in Mottled Ducks.” Wilson Bulletin 108, no. 1: 187–189.

[jmor70027-bib-0016] Koecke, H.‐U. 1958. “Normalstadien Der Embryonal‐Entwicklung Bei Der Hausente (Anas Bosch as Domestic a).” Embryologia 4, no. 1: 55–78. 10.1111/j.1440-169x.1958.tb00147.x.

[jmor70027-bib-0017] Li, S. , S. Bai , X. Qin , et al. 2019. “Comparison of Whole Embryonic Development in the Duck (*Anas platyrhynchos*) and Goose (*Anser cygnoides*) With the Chicken (*Gallus Gallus*).” Poultry Science 98, no. 8: 3278–3291. 10.3382/ps/pez133. 30941418

[jmor70027-bib-0018] Louryan, S. , M. Choa‐Duterre , G. Destoop , M. Lejong , C. Matte‐Allain , and N. Vanmuylder . 2023. “Development of the Goose Tongue Filiform Papillae: Could It Be Tooth‐Like Sense Organs?” International Journal of Morphology 41, no. 6: 1631–1639.

[jmor70027-bib-1005] Lukaszewicz, E. , M. Lason , J. Rosenberger , A. Kowalczyk , and M. Bakst . 2017. “Goose Embryonic Development From Oviposition Through 16 Hours of Incubation.” Poultry Science 96, no. 6: 1934–1938. 10.3382/ps/pew474. 28053196

[jmor70027-bib-0019] Maxwell, E. E. 2008. “Ossification Sequence of the Avian Order Anseriformes, With Comparison to Other Precocial Birds.” Journal of Morphology 269, no. 9: 1095–1113. 10.1002/jmor.10644. 18496857

[jmor70027-bib-0020] Mizia, P. C. , I. Rams‐Pociecha , E. Podmokła , and R. P. Piprek . 2023. “Histological Analysis of Early Gonadal Development in Three Bird Species Reveals Gonad Asymmetry From the Beginning of Gonadal Ridge Formation and a Similar Course of Sex Differentiation.” Annals of Anatomy‐Anatomischer Anzeiger 250: 152151. 10.1016/j.aanat.2023.152151 37574173

[jmor70027-bib-0021] Montgomery, R. A. , C. J. Burke , and S. M. Byers . 1978. “A Field Guide to the Aging of Wood Duck Embryos.” Journal of Wildlife Management 42, no. 2: 432. 10.2307/3800287.

[jmor70027-bib-0022] Ran, M. , Q. Ouyang , X. Li , et al. 2023. “Exploring Right Ovary Degeneration in Duck and Goose Embryos by Histology and Transcriptome Dynamics Analysis.” BMC Genomics 24, no. 1: 389. 10.1186/s12864-023-09493-0. 37430218 PMC10332064

[jmor70027-bib-1006] Ricklefs, R. E. , and J. M. Starck . 1998. “Embryonic Growth and Development.” In Avian Growth and Development, edited by J. M. Starck and R. E. Ricklefs , 31–58. Oxford, UK: Oxford University Press.

[jmor70027-bib-1002] Stern, C. D. 2018. “Staging Tables for Avian Embryos: A Little History.” International Journal of Developmental Biology 62, no. 1–2–3: 43–48. 10.1387/ijdb.170299cs. 29616737

[jmor70027-bib-0023] Stunden, C. , C. Bluhm , K. Cheng , and R. Rajamahendran . 1998. “Plasma Testosterone Profiles, Semen Characteristics, and Artificial Insemination in Yearling and Adult Captive Mallard Ducks (*Anas platyrhynchos*).” Poultry Science 77, no. 6: 882–887. 10.1093/ps/77.6.882. 9628539

[jmor70027-bib-0024] Xu, R. F. , W. Wu , and H. Xu . 2007. “Investigation of Feather Follicle Development in Embryonic Geese.” Poultry Science 86, no. 9: 2000–2007. 10.1093/ps/86.9.2000. 17704390

[jmor70027-bib-0025] Yamasaki, M. , and A. Tonosaki . 1988. “Developmental Stages of the Society Finch, *Lonchura striata* Var. Dornestica. (Society Finch/Developmental Stages/Morphogenesis/Laboratory Animal).” Development, Growth & Differentiation 30, no. 5: 515–542. 10.1111/j.1440-169x.1988.00515.x. 37281331

[jmor70027-bib-0026] Zou, H. , and L. Niswander . 1996. “Requirement for BMP Signaling in Interdigital Apoptosis and Scale Formation.” Science 272, no. 5262: 738–741. 10.1126/science.272.5262.738. 8614838

