# Supplementary material for: Morphological Criteria for Staging Near‐Hatching Embryos of the Domesticated Mallard (Anas platyrhynchos) and Swan Goose (Anser cygnoides)
Source: J Morphol. 2025 Jan 29;286(2):e70027. doi: 10.1002/jmor.70027 (PMC11780221; doi:10.1002/jmor.70027)
Supplement: Supplementary file 1 — Supporting information. [file JMOR-286-e70027-s002.docx]

**
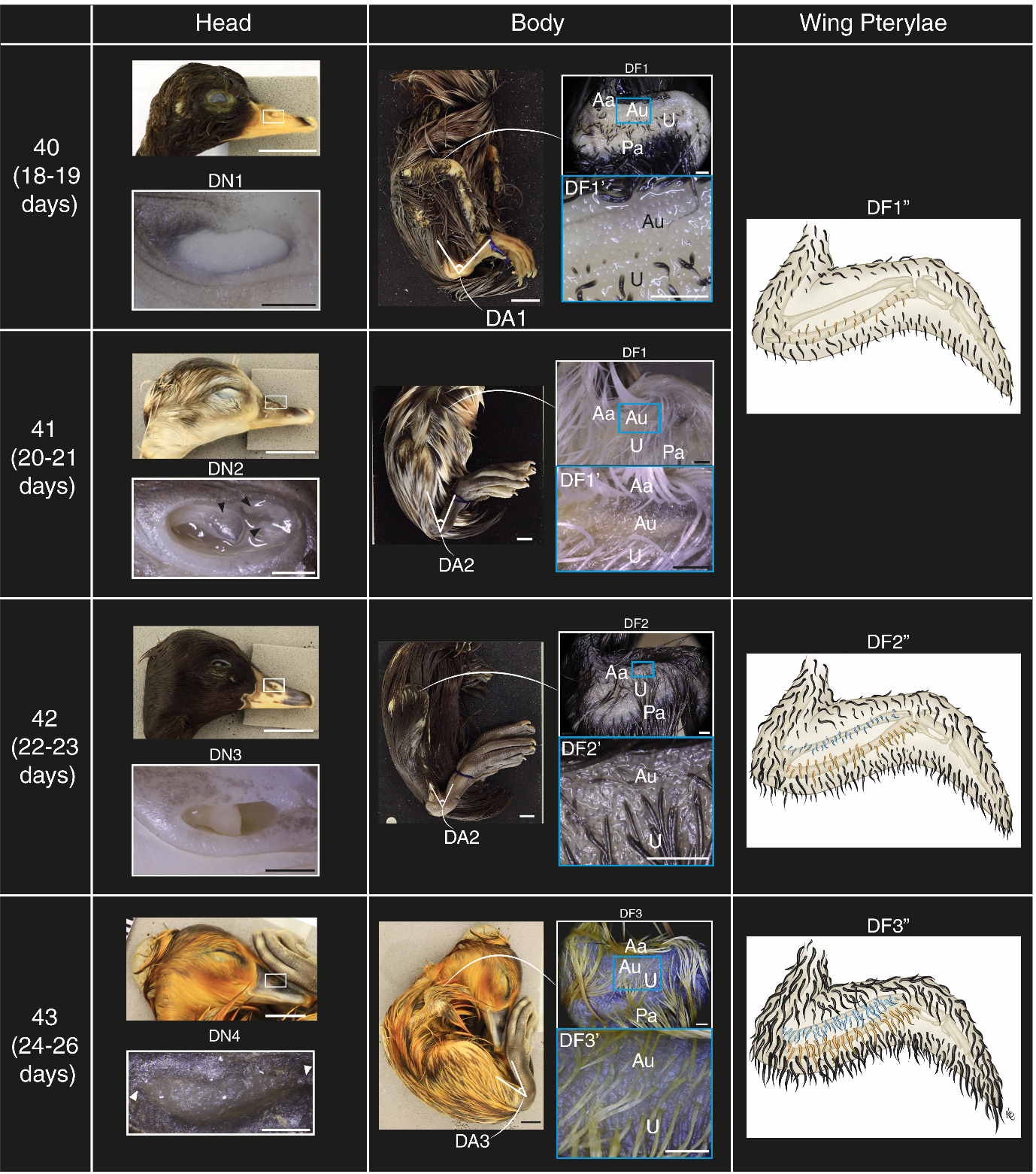
**Supplementary Figures

**Figure S1: Staging criteria for near-hatching embryos of the Mallard duck (*Anas platyrhynchos*).** DN (1-4): Phases of nostril development. DN2: In the second phase of nostril development four nasal components appear, indicated by black arrowheads. DN4: The narrow rostral and caudal ends are indicated by white arrowheads. DA (1-3): phases of changes in angle of ankle flexure. DF (1-3): Phases of feather tract development. DF1’: inset showing the absence of anterior ulnoradial tract at stages 40 and 41. DF2’: inset showing a short anterior ulnoradial tract at stage 42. DF3’: inset showing a longer anterior ulnoradial tract at stage 43. DF (1”-3”): illustrations of the phases of feather tract development. The anterior ulnoradial tract is shown in blue, and the ulnoradial tract is shown in orange. DA (1-3): Phases of ankle development. Embryo at stage 43 died during hatching and, it was extracted, preserved and photographed as a whole body. Grey scale bars are 1 cm and inset scale bars are 1 mm. Abbreviations: Aa; anterior alar, Au; anterior ulnoradial, U; ulnoradial, Pa; posterior alar tracts.


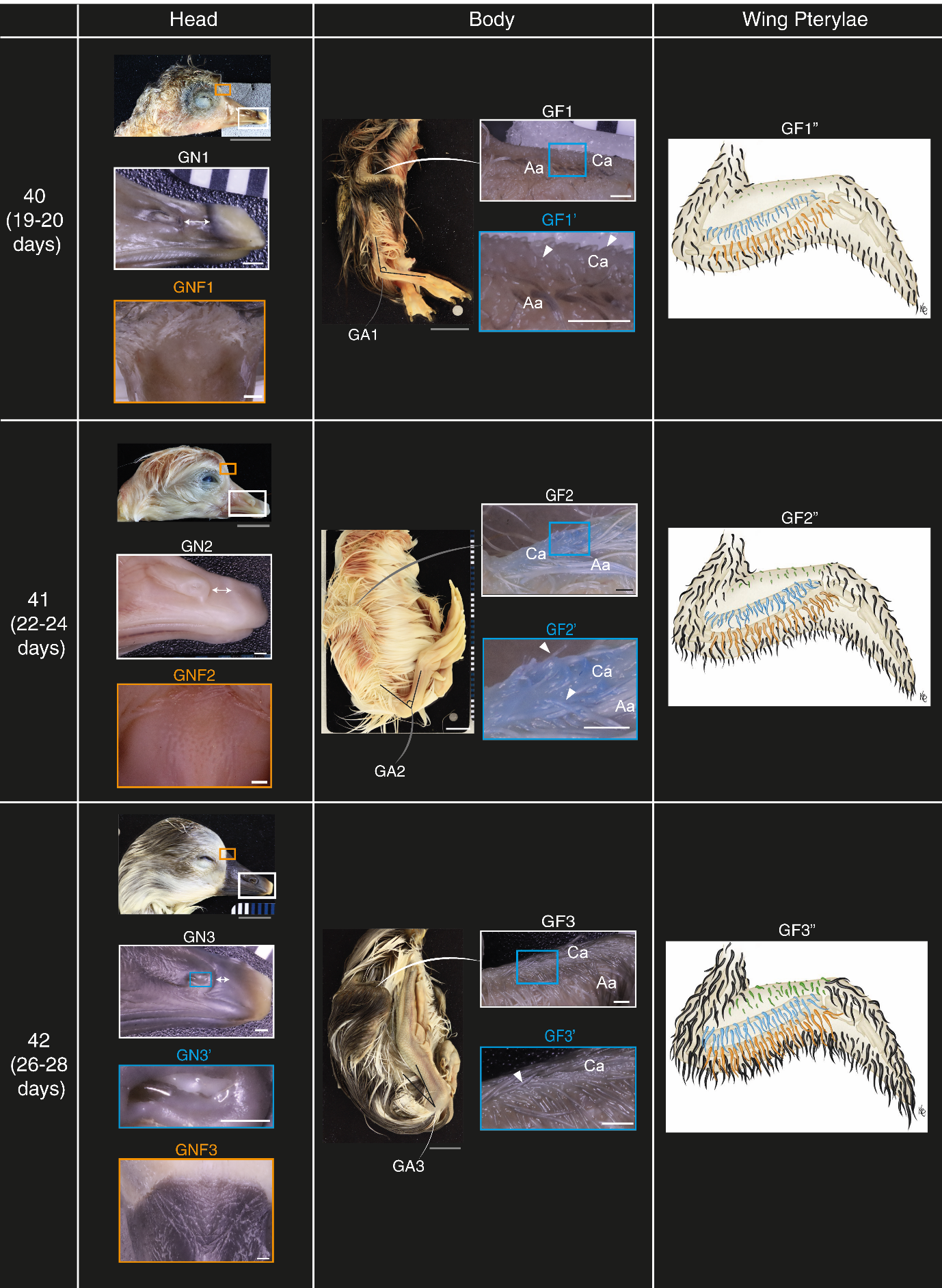
**Figure S2: Staging criteria for near-hatching embryos of Swan Goose (*Anser cygnoides*)** GN (1-3): Phases of nostril development. In the first and second phases the distance between the nostril and the egg tooth is indicated with a double headed arrow (GN1-2). GN3: At the third phase a pointed white nasal component appears. GNF (1-3): Phases of nasofrontal hinge development. GNF2: One of the deep pits of the second phase of nasofrontal hinge is indicated by a white arrowhead. GF (1-3): Phases of cranial feather tract development; tracts are indicated with black arrowheads. GF (1”-3”): illustrations of the phases of feather tract development. The cranial alar tract is shown in green, anterior ulnoradial tract is shown in blue, and the ulnoradial tract is shown in orange. GA (1-3): Phases of ankle development. Grey scale bars are 1 cm and inset scale bars are 1 mm. Abbreviations: Aa; anterior alar, Ca; cranial alar.
